# Clonogenic Multiple Myeloma Cells have Shared stemness Signature Associated with Patient Survival

**DOI:** 10.18632/oncotarget.1145

**Published:** 2013-07-15

**Authors:** Renji Reghunathan, Chonglei Bi, Shaw Cheng Liu, Koh Tze Loong, Tae-Hoon Chung, Gaofeng Huang, Wee Joo Chng

**Affiliations:** ^1^ Department of Medicine, National University of Singapore, Singapore; ^2^ Cancer Science Institute, National University of Singapore, Singapore; ^3^ SIRTex, 50 Science Park Road, Singapore Science Park II, Singapore; ^4^ Department of Haematology-Oncology, National University Hospital, National University Health System, Singapore

**Keywords:** myeloma, plasma cells, proliferation, differentiation, stemness

## Abstract

Multiple myeloma is the abnormal clonal expansion of post germinal B cells in the bone marrow. It was previously reported that clonogenic myeloma cells are CD138^−^. Human MM cell lines RPMI8226 and NCI H929 contained 2-5% of CD138^−^ population. In this study, we showed that CD138^−^ cells have increased ALDH1 activity, a hallmark of normal and neoplastic stem cells. CD138^−^ALDH^+^ cells were more clonogenic than CD138^+^ALDH^−^ cells and only CD138^−^ cells differentiated into CD138^+^ population. *In vivo* tumor initiation and clonogenic potentials of the CD138^−^ population was confirmed using NOG mice. We derived a gene expression signature from functionally validated and enriched CD138^−^ clonogenic population from MM cell lines and validated these in patient samples. This data showed that CD138^−^ cells had an enriched expression of genes that are expressed in normal and malignant stem cells. Differentially expressed genes included components of the polycomb repressor complex (PRC) and their targets. Inhibition of PRC by DZNep showed differential effect on CD138^−^ and CD138^+^ populations. The ‘stemness’ signature derived from clonogenic CD138^−^ cells overlap significantly with signatures of common progenitor cells, hematopoietic stem cells, and Leukemic stem cells and is associated with poorer survival in different clinical datasets.

## INTRODUCTION

Multiple myeloma (MM) is a post-germinal B-cell malignancy characterized by accumulation of clonal plasma cells in the bone marrow, which secrete abnormal monoclonal proteins [1]. Genetic aberrations such as gains and losses of whole chromosomes, by non-random chromosomal translocations causing dysregulation of genes at breakpoints, and by point mutations, have been implicated in disease pathogenesis and progression [2, 3]. In spite of therapeutic advances in the last decades, myeloma is still generally incurable characterized by recurrent relapse following a period of remission to different therapeutic regimen.

Accumulating evidence has proposed a model in which tumorigenesis is driven by cancer stem cells (CSC) that are derived from mutated adult stem cells [4]. CSCs undergo self renewal, recapitulate the phenotype of the tumor from which they were derived, develop into phenotypically diverse cancer cell populations, proliferate extensively, and drive both continued expansion of malignant cells and resistance to chemotherapy. It has been postulated that these rare population are inherently drug resistant, and also the source of subsequent relapse. Recent studies suggest myeloma may follow a similar model. The majority of myeloma plasma cells appear quiescent and the potential for clonogenic growth may be limited to a minority population of cells, that are resistant to therapy and mediate subsequent relapse [5]. Indeed, recent studies have shown that multiple myeloma consists of heterogeneous cell types and with different clonogenic potential [6]. It was demonstrated that the clonogenic myeloma ‘*stem cells*’ have an immunophenotype similar to memory B-cells [7, 8]. These cells exhibit properties of stems cells and are found to be resistant to treatment by current active compounds used in the treatment of the disease [9, 10]. Syndecan-1 (CD138) is expressed by malignant plasma cells from the majority of MM cell lines and patient specimens. It is highly specific for terminally differentiated normal plasma cells and is absent on highly proliferative normal plasmablasts and all earlier B-cell stages [11-13]. It has been found that, human MM cell lines and clinical specimens contained small (< 5%) subpopulations that lacked CD138 expression [6, 14]. These CD138^−^ cells have greater clonogenic potential *in vitro* and *in vivo* than CD138^+^ plasma cells and exhibit stem cell properties that mediate drug resistance [9, 15]. Recently, many researchers are focusing on these myeloma stem cells and their involvement in myeloma initiation and relapse. However, the exact mechanism and their functional roles in the disease process are yet to be explored. A thorough understanding of the molecular signature of the clonogenic population may unravel their biological roles in myeloma as well as identify potential new therapeutic avenues to eradicate these drug-resistant populations. Furthermore, the presence of these populations and hence this molecular signature may identify subset of patients with different clinical outcome. In this study, we generated a gene expression signature from functionally validated and enriched CD138^−^ clonogenic population from human myeloma cell lines and validated this in patient samples. This signature was enriched for previously identified genes, expressed in benign and malignant stem cells and when applied to clinical myeloma dataset was highly correlated with survival, substantiating a major prediction of the CSC model in multiple myeloma.

## RESULTS

### Human myeloma cell lines contained about 2-5% of CD138^−^ population that has increased aldehyde dehydrogenase (ALDH) enzyme activity.

**Figure 1:**
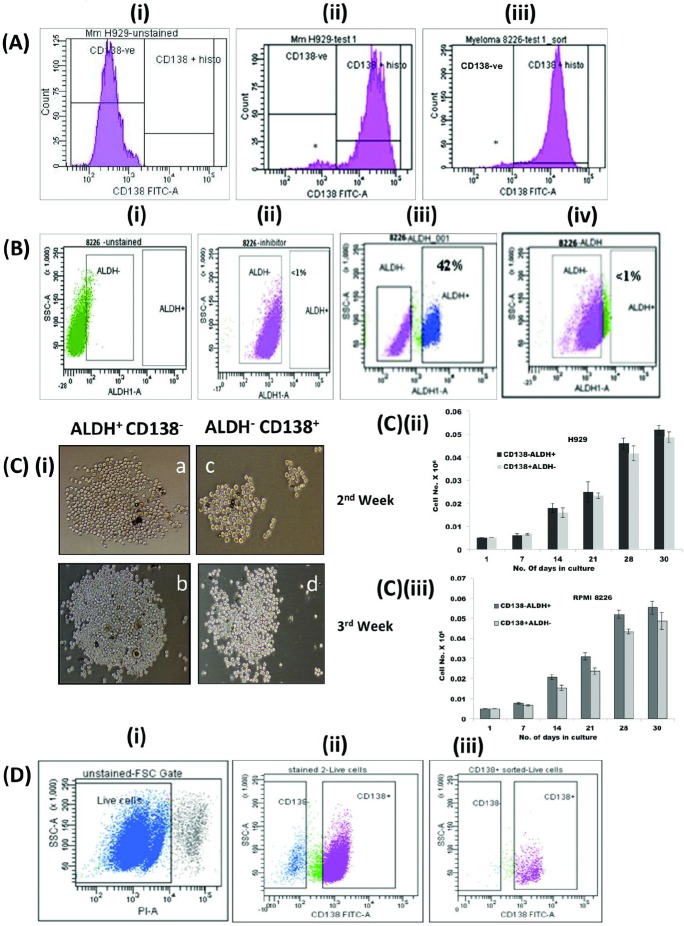
Properties of clonogenic population of myeloma cells (A) Human MM cell lines H929 and RPMI 8226 contained 2-5% of CD138^−^ population. Flow cytometric analysis of (i) unstained control cells (H929), (ii) CD138 FITC antibody treated H929 and (iii) RPMI8226 cells. *denotes CD138^−^ population. (B) About 42% of CD138^−^ population from myeloma cells displayed increased ALDH1 activity. CD138^+^ and CD138^−^ subsets of RPMI 8226 cells were treated with aldefluor reagent, with or without DEAB inhibitor and ALDH1 activity was measured by flow cytometry. Flow cytometric analysis of (i) untreated control cells, (ii) cell treated with DEAB inhibitor and aldefluor reagent, (iii) aldefluor reagent treated CD138^−^ and (iv) CD138^+^cells. (C) CD138^−^ ALDH^+^ cells were more clonogenic than CD138^+^ ALDH^−^ cells on methylcellulose medium. CD138^−^ALDH^+^ and CD138^+^ALDH^−^ cells were cultured in growth medium containing methylcellulose for 3-4 weeks and their colony forming potential was assessed [6]. Pictures on panel (i) depict morphology of the colonies of CD138^−^ ALDH^+^ (a, b) and CD138^+^ ALDH^−^ cells (c, d) on MC medium on 2^nd^ and 3^rd^ week respectively. C (ii) p<0.03 and C (iii) p<0.03 are graphical representation of their clonogenicity. The experiments were conducted in triplicates. Correction bar represents SD. *p-*values were calculated by paired Student's *t* test across the timepoints: 2-tailed *p*-value <0.03 and <0.02 for RPMI and H929 respectively. (D) CD138^−^ cells differentiated into CD138^+^cells, while CD138^+^cells lost their ability to differentiate on long term culturing. (i) Flow cytometric analysis of cells grown from CD138^−^ cells showed two distinct population on forward scattering, (ii) cells grown from CD138^−^ cells differentiated into CD138^+^ and CD138^−^ populations (iii) while CD138^+^ cells remained as CD138^+^.

Consitent with previous reports [6,9,10] human MM cell lines RPMI8226 and NCI-H929 contained distinct subset of CD138^−^ cells that represent about 2-5 % of the total population (Fig 1A). When assessed by the Aldeflour assay, about 42% of the CD138^−^ cells (0.5-1.3 % of the total population) were ALDH^+^ while CD138^+^ cells have less than 1% of ALDH^+^ population (Fig 1B). Increased expression of ALDH1 enzyme is an established property of stem cells from MM, lung cancer, acute myeloid leukemia, brain and breast cancers [9, 15, 16-20].

### CD138^−^ ALDH^+^ cells were more clonogenic than CD138^+^ALDH^−^ cells when cultured in methylcellulose (MC) medium

To assess clonogenicity, ALDH^+^ CD138^−^ and ALDH^−^ CD138^+^ populations of myeloma cells were plated onto MC medium and allowed to grow for 4 weeks and their colony forming potential was assessed. During the culture, CD138^−^ ALDH^+^ cells were found to be more proliferative and produced larger colonies compared to CD138^+^ ALDH^−^ cells, though CD138^−^ALDH^+^ population took more days to produce initial colonies. However, on subsequent serial plating CD138^−^ALDH^+^ cells showed significantly greater clonogenic expansion (paired student t-test p-value of <0.03 and <0.02 for RPM1 and H929 respectively), although the absolute differences are relatively small (Fig 1C). Upon long-term culture, CD138^−^ cells, but not the CD138^+^ cells, produced both CD138^+^ and CD138^−^ populations, confirming the ability of the clonogenic CD138^−^ cells to recapitulate myeloma with the bulk population of CD138^+^ cells and fewer CD138^−^ cells (Fig 1D).

### Assessment of in vivo clonogenicity and tumor initiation in NOG mice

**Figure 2:**
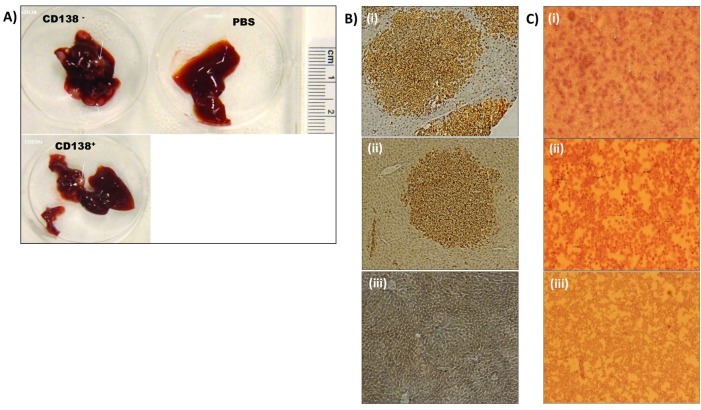
CD138- cells colonized and produced tumors in the liver and bone marrow of mice NOG mice injected with CD138- or CD138+ cells or PBS into their tail vein and observed for 14-16 weeks were sacrificed once they developed symptoms of myeloma or at the end of the observation period; liver and bone marrow were collected from them. (A) Liver tissues harvested from mice injected with (i) CD138^−^, (ii) CD138^+^ cells but not (iii) PBS developed tumor nodules (arrows). However tumor frequency was less and tumor size was smaller in those injected with CD138^+^ cells. (B) Human CD138^+^ cells were detectable by immnohistochemistry in the liver tumor of mice injected with (i) CD138^−^ and (ii) CD138^+^ cells. CD138^+^ cells are not detectable in liver tissue of mice injected with (iii) PBS. (C) Immuno-detection of human CD138^+^ cells (arrows) in the bone marrow of mice injected with (i) CD138^−^, (ii) CD138^+^ but not those injected with (iii) PBS. Original magnification (Olympus CK 40 microscope): X100 for panels B and C.

We performed *in vivo* clonogenic and tumor initiation experiments in NOG mice using the clonogenic population isolated from the MM cell lines. CD138^−^ cells produced tumor in all six mice whereas CD138^+^ cell were able to produce tumor in only two out of six mice (Table 1), further suggesting the greater clonogenic and tumor initiating potential of CD138^−^ population. Detection of human CD138^+^ cells in the tumor tissues of liver and bone marrow harvested from these mice confirmed that the tumors originated from the injected cells (Fig 2). These studies established that the clonogenic cells are enriched in the CD138^−^ population.

**Table 1:** Comparison of tumor initiation and engraftment potential of CD138^−^ and CD138^+^ subsets in NOG mice

Cell type injected	No. of mice with posterior leg paralysis	No. of mice showed human CD138^+^ cells in liver and bone marrow on IHC
CD138−	3/6	6/6
CD138+	2/6	2/6
PBS	0/6	0/6

### Gene expression profiling (GEP) of CD138^+^ versus CD138^−^ population identifies a signature of proliferation and self-renewal in the CD138^−^ population

In order to understand the molecular regulation of stemness in the CD138^−^ subset, we subjected each functionally validated CD138^−^ and CD138^+^ fractions from both cell lines to global gene expression analysis (GEO Accession No: GGSE31305). There were 113 differentially expressed genes (2-fold or greater difference and in the same direction) in CD138^+^ cells compared to CD138^−^ cells common to both the cell lines (Supplementary Table S1). Genes involved in cell proliferation (*STAG2, RB1CC1* etc), Polycomb Repressor Complex (PRC) genes that regulate proliferation and differentiation *(BMI1, SUZ12, EZH2* etc), signal transducers (*RAB18, SHOC2* etc), and regulators of transcription and translation (*ZNF146, EIF1AX* etc) were differentially expressed.

**Figure 3:**
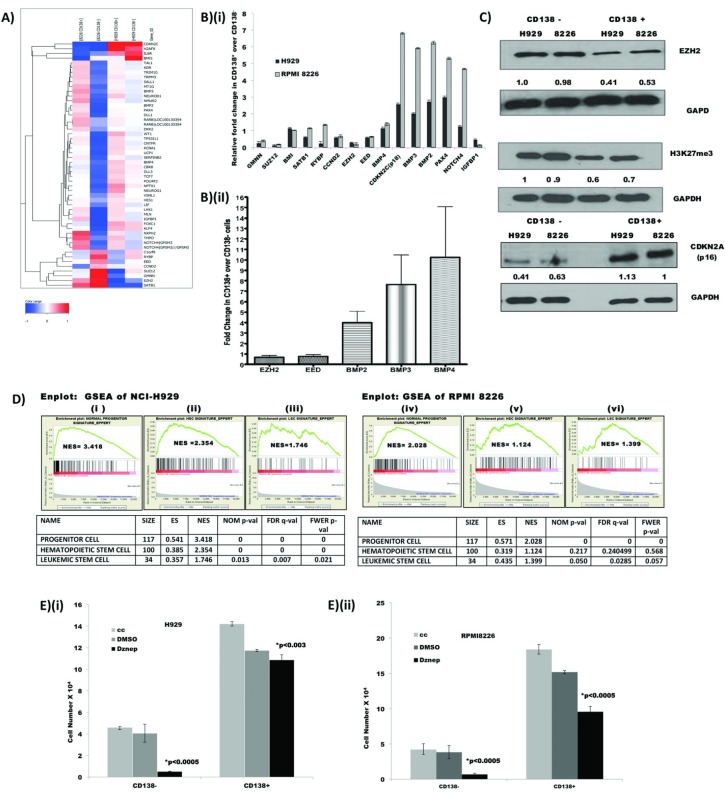
GEP showed differential expression of polycomb repressor complex (PRC) and their target genes (A) Heatmap depicting differential expression of PRC genes and their targets [37, 38, 41] in CD138+ and CD138- MM cell populations. Genes with a fold change of 1.3 or more, in at least one of the cell lines were selected to create the Heatmap; as EED is an important PRC2 gene, it was included in the list though the fold change was less than 1.3 in both the cell lines (Supplementary table S2). (B) Validation of selected PRC genes and their targets by q-PCR: fold change of CD138^+^ over CD138^−^ was calculated by 2ddC_T_ method [51] after normalizing against GAPDH in both (i) cell lines and (ii) patient samples. If the relative fold change is less than 1.0, the expression of the gene is higher in the CD138^−^ cells and vice versa. In patient samples, fold change of each transcript is the mean value of five patients and error bar represents the SD, (Primer list in supplementary Table S4). (C) Western blot analysis of CD138^−^ and CD138^+^ cells from myeloma cell lines H929 and RPMI 8226 showing over expression of PRC protein EZH2 in CD138^−^ cells and subsequent upregulation of its histone target H3K27me3 and down-regulation of CDKN2A(p16). The numbers below each panel depict the relative quantification of their expression after normalizing against GAPDH using IMAGE J. (D) CD138^−^ cells displayed shared expression profile with PC, HSC and LSC: Enplots showing enrichment of common progenitor cell (PC) signature, haematopoetic stem cell (HSC) signature, and Leukemic stem cells (LSC) signature in H929 (i-iii) and RPMI 8226 (iv-vi) cells. NES denotes normalized enrichment score. Heatmap of the lead genes have been depicted in Supplementary Figure S1. (E) Sensitivity of CD138^−^ and CD138^+^ cells to DZNep treatment: CD138^−^ and CD138^+^ cells were plated onto 96-well plate in triplicates and treated with 0.5μM/L of DZNep or 0.1% of DMSO for 48 hrs and the cell viability was assessed. The graph depicts the number of viable cells in each group after the incubation period and bars represent the SD and *p-*values were calculated by Student's *t* test (*p value for comparison between DMSO treated and DZNep treated samples).

As PRC genes play a pivotal role in cell fate determination, we further examined the GEP data using the less stringent criteria of 1.3 fold difference in expression in at least one of the cell lines and found that there was a coordinated differential expression of more PRC proteins and their targets in the CD138^−^ compared to CD138^+^ population (Fig 3A and Supplementary Table S2). Transcripts of PRC2 proteins EZH2, EED and SUZ12 as well as PRC1 component BMI1 were upregulated in the clonogenic population compared to CD138^+^ cells. The PRC methylates histones and represses transcription of its target genes [21]. Consequently, their targets like cyclin dependent kinase inhibitors (*CDKN1A*, *CDKN1C*, *CDKN2A*, *CDKN2C* etc) and differentiation promoting genes (*BMP2*, *BMP3*, *BMP9, PAX4, NEUROD* etc) were significantly down regulated. The decreased expression of CDKN1A (p21) and CDKN1C (p57), may lead to the upregulation of CDK2 and CDK4 expression in CD138^−^ cells, and the subsequent over-expression of cell division cycle 2 (CDC2) and other cyclins, along with cyclin related proteins may drive the CD138^−^ cells to enter into G1/S transition and G2/M phase of cell cycle and proliferate [22] (Supplementary Table S3 lists differentially expressed cell cycle associated genes in CD138^+^ and CD138^−^ populations). Differential expression of PRC genes, their selected targets and cell cycle associated genes were validated by q-PCR (Fig 3B (i)). We further validated the GEP findings in cell lines on five human myeloma patient samples by qPCR of selected genes (Fig 3B (ii)). Transcripts of PRC2 proteins such as EZH2, EED were down-regulated and proteins that favour differentiation, such as BMP2, BMP3 and BMP4 were upregulated in CD138^+^ populations compared CD138^−^ cells, consistent with the findings in the cell lines. As EZH2 has recently been implicated in a number of haematological malignancies [23-27], we examined the potential functional relevance of increased EZH2 expression in CD138^−^ myeloma cells. Using western blot, we confirmed that EZH2 protein is also highly expressed in CD138^−^ cells. We further showed that, in CD138^−^ cells, there is more trimethylation of the repressive H3K27 histone mark (H3K27me3) with consequent repression of one of the established target genes whose expression is regulated by EZH2, CDNK2A[28,29] (Fig 3C). Taken together the differential expression of PRC proteins in clonogenic population of myeloma cells may lead to the epigenetic silencing of many PRC target genes regulating differentiation or senescence and driving the cells to cycle and self-renew.

### CD138^−^ cells share a molecular profile with common progenitor cells (PC), hematopoietic stem cells (HSC) and leukemic stem cell (LSC).

To further verify that the gene expression signature of CD138^−^ reflects a transcription program of self-renewal and ‘stemness’, we performed Geneset Enrichment Analysis (GSEA) to examine the extent of shared expression pattern between the gene expression signature of CD138^−^ cells compared to CD 138^+^ cells with three other published gene sets representing PC, HSC and LSC signatures. The CD138^−^ GE profile was significantly enriched for genes constituting the PC, HSC and LSC signatures (Fig 3D), suggesting that LSC, HSC and PC expression programs are preferentially expressed in clonogenic CD138^−^ cells compared to the more mature CD138^+^ myeloma cells (supplementary Figure S1). The gene expression profile of CD138^−^ cells therefore contained functional expression modules consistent with the clonogenic phenotype of CD138^−^ myeloma cells.

### CD138^−^ cells were more sensitive to DZNep treatment

DZNep(3- Deazaneplanocin), a compound that disrupts the PRC2, effectively depletes EZH2, Suz12, and EED and inhibits H3K27me3 in breast cancer and AML cells [30-32]. As inhibition of EZH2 and H3K27me3 may affect clonogenicity and differentiation in normal stem cells and the GEP and western blot results implicated a central role for PRC2 proteins in the self-renewal of clonogenic myeloma cells, we wanted to examine whether DZNep, exerted a differential effect between CD138^−^ and CD138^+^ populations. Indeed CD138^−^ cells were more sensitive than CD138^+^ cells to DZNep treatment (Fig 3E). This suggests that CD138^−^ cells are more dependent on PRC2 proteins for their survival and self-renewal. This finding is consistent with the gene expression results and similar to previous reports in AML stating DZNep targets primarily leukemic stem cell (CD34^+^CD38^−^) rich population [32].

### CD138^−^ cell signature predicts patient survival

**Figure 4:**
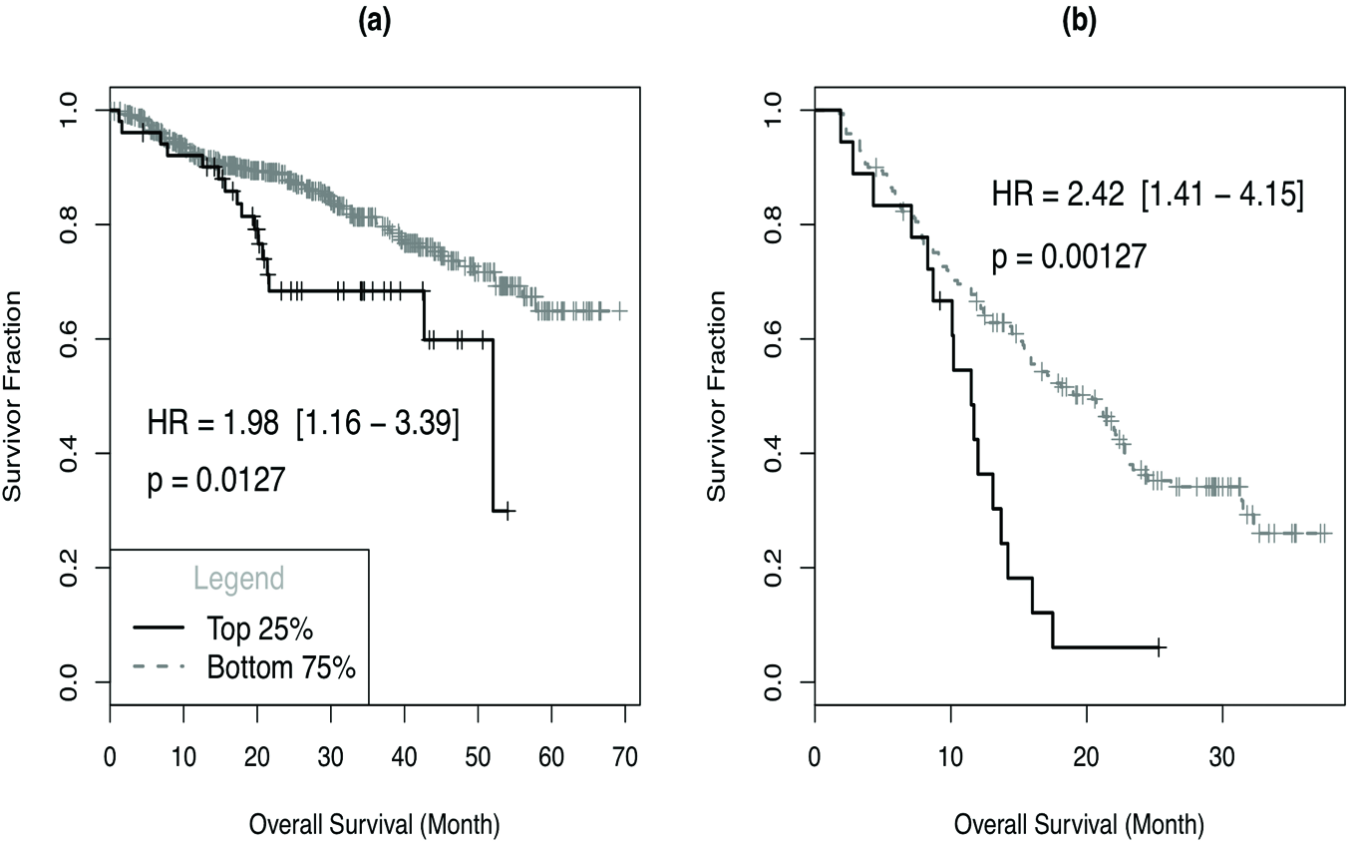
Gene signature of CD138- population is correlated with patient outcome MAS5 processed gene expression profiling data for UAMS dataset (GSE2658) and Bortezomib dataset (GSE9782) were normalized with probeset-wise median normalization; for each probeset, we first determined its median MAS5 intensity level over all samples and expression value is estimated by the logarithm (base 2) of MAS5 intensity divided by median level (log2ratio). Expression measure of a sample was calculated as the median of log2 ratio for signature probesets. Survival association was assessed by the Cox proportional hazard regression analysis using signature groups obtained by dividing signature index values into 4 equally spaced segments across the whole range and plotted survival factor (y-axis) against months (x -axis) in case of comparison with (a) UAMS and (b) bortezomib datasets. The number of samples in top quartile and the bottom 3 quartile are as follows: UAMS dataset: 51 (top 25%), 508 (bottom 75%); Bortezomib dataset: 18 (top 25%), 170 (bottom 75%).

We investigated the clinical relevance of the clonogenic signature derived from myeloma cell lines in two clinical data sets - UAMS dataset (GSE2658) and Bortezomib dataset (GSE9782). A high expression of the clonogenic signature is associated with significantly poorer survival in both newly diagnosed patients who were on the transplant-based total therapy and relapse patients treated with bortezomib (Fig 4 a,b). This is consistent and provides further verification for the presence of clonogenic signature that may portend drug resistance and relapse in myeloma.

## DISCUSSION

Many cancers are hierarchically organized and sustained by a subpopulation of clonogenic cells that have self renewal capacity. Tumor re-growth following initial reductions in disease burden suggests that tumor cells capable of clonogenic growth are relatively drug resistant, and in several human cancers these functional properties have been attributed to cancer stem cells. Multiple myeloma is a neoplastic clonal expansion of phenotypically heterogeneous population of plasma cells that secrete abnormal amounts of monoclonal antibodies. Terminally differentiated CD138^+^ plasma cells constitute the majority of the tumor cells in MM but are unable to sustain the clonogenic growth indefinitely. Chemoresistant self-renewing population resembling memory B cells isolated from myeloma patient samples and cell lines have been reported earlier [9]. These cells demonstrated functional properties similar to normal stem cells suggesting that specific cellular processes regulating normal stem cells may be active within CSC and serve as potential therapeutic targets. Although highly clonogenic cell populations have been identified in myeloma that are able to phenotypically recapitulate the original tumor in immune compromised mice, the clinical relevance and implications of these findings remain unclear.

In our study, we isolated clonogenic CD138^−^ cells from two myeloma cell lines RPMI 8226 and NCI H929 which were used to generate xenograft in NOG mice and for gene expression studies. CD138^−^ cells showed 100% tumor initiation and disease progression in mice (six out of six) as evident from the clinical signs and presence of human CD138^+^ cells in their liver and bone marrow on immnohistochemistry analyses. The CD138^−^ cells injected could initiate tumor development, and differentiate into CD138^+^ cells, confirming our *in vitro* observation. Only two out of six mice injected with CD138^+^ cells showed tumor initiation and the extent of tumor development was much less in these two mice. The exact reason for the tumorigenicity by CD138^+^ cells is unclear, but there are recent reports, by Fuhler et al demonstrating a reduced expression of syndican-1 (CD138) and a change in kinome profile of CD138^+^ cells, when they interacted with bone marrow stromal cells [33] and by Chaidos et al confirming the existence of an interconvertible tumor propagating population of CD138^+^ and CD138^−^ cells that are controlled by epigenetic regulators and linked to drug resistance in myeloma patients [34]. So it is plausible that the injected CD138^+^ cells in mice might have reduced the expression of CD138 on their membrane surface and acquired properties similar to CD138^−^ population when they interacted with the bone marrow stromal cells *in vivo.* The CD138^−^ population has the essential set of biological functions common to all stem cells, including self-renewal, increased activity of ALDH1 and the ability to produce differentiated, less clonogenic CD138^+^ mature plasma cells. Clonogenic CD138^−^ cells were heterogeneous and included different stages of B cell differentiation[35], (Supplementary Fig S2) suggesting that the clonogenicity and tumorigenicity of CD138^−^ population were an attribute of a heterogeneous population rather than of a single cell type.

Gene expression profiling of well-defined clonogenic and non-clonogenic populations helped to identify bona fide CSC-specific signature on a genome-wide basis. Polycomb repressor complexes (PRC), their target proteins and cell cycle associated genes constituted majority of differentially expressed genes. PRC are regulatory proteins that collaborate to exert transcriptional repression of target genes by gene specific recruitment [36]. Upregulation of PRC2 components *EZH2*, *EED* and *SUZ12* result in down regulation of their target gens like cyclin dependent kinase inhibitors *CDKN1A* (*p21*), *CDKN2A* (*p16*), *CDKN2C* (*p18*) and *CDKN1C* (*p57*), which in turn may upregulate the expression of cyclin dependent kinases and cyclins (supplementary Table S3), together with the downregulation of differentiation promoting genes (*BMP2*, *BMP3*, *BMP4*, *PAX4*, *NOTCH4* etc) [37-41]. We observed these predicted changes in our gene expression results providing a molecular basis for clonogenic CD138^−^ cells to proliferate and self renew, instead of terminally differentiating into CD138^+^ plasma cells. This observation is strengthened by the significant overlap in the gene expression profile of clonogenic myeloma cells derived in our study and the published transcription profiles of PCs, HSCs and LSCs.

Recently, methyltransferase inhibitor DZNep has been reported to decrease PRC2 proteins, including EZH2, and trigger caspase mediated apoptosis in myeloma cells [42]. The differential effect of DZNep on CD138^−^ and CD138^+^ cells suggest that the clonogenic cells are dependent on higher levels of EZH2 and other PRC2 proteins to survive and self renew, and these molecules may be potential therapeutic targets in this population of cells. This is particularly of clinical relevance as drugs targeting EZH2 are currently in development [28, 43-46]. Validation of key genes in the clonogenic signature in clinical samples suggests that the molecular differences detected in cell lines are also relevant to patients. Indeed, myeloma with higher expression of the clonogenic transcriptional signature have significantly poorer survival, suggesting that the transcriptional program governing ‘stemness’ in myeloma has an influence on clinical outcomes.

Overall, our data provide evidence of molecular pathways underlying clonogenic cells in myeloma and their clinical relevance and offer potential insights into pathways that can be targeted for treatment.

## MATERIALS AND METHODS

### Cell lines and Cell culture

Human MM cell lines RPMI8226 and NCI-H929 were cultured in complete media consisting of RPMI1640, 2mM L glutamine, 50U/ml of Penicillin, 50 μg /ml of Streptomycin and 10% of fetal bovine serum (FBS). Syndican-1 (CD138) positive and negative subsets were separated using antihuman CD138 antibody conjugated with FITC (BD PharMingen) by flow cytometry (BD FACSaria II). Clonogenic growth was evaluated by plating 1000 cells/ml in 1.2% methyl cellulose medium as described by Matsui et al [6]. Cells were plated in quadruplets onto 35mm tissue culture dishes and incubated at 37° C and 5% CO_2_. Colonies consisting of more than 40 cells were scored. Serial replating was performed by washing plates 3 times with complete media and resuspending cells into original volume of methylcellulose as above.

### Flow cytometry

Cell were initially depleted of necrotic cells by density centrifugation then stained with FITC conjugated mouse antihuman CD138 antibodies (BD PharMingen™, BD Biosciences, NJ, USA) for 30 minutes at 4°C. Cells were washed then resuspended in phosphate buffered saline (PBS) containing 5μM propidium iodide (Sigma-Aldrich, MO, USA) and sorted on BD FACSaria™ II (BD Biosciences, NJ, USA) sorter. Cells were gated to exclude PI^+^ cells and sorted into CD138^−^ and CD138^+^ fractions by gating on the lowest and highest 5%FITC expressing cells respectively [6]. Following sorting, the CD138^+^ and CD138^−^ cells fractions were analyzed by flow cytometry and found to be more than 98% pure. For phenotype analyses, cells were prepared as described above and stained with CD138 FITC, CD27 PE and CD19 APC or isotype control antibodies (BD PharMingen, ™ BD Biosciences, NJ, USA). Cells were analyzed by gating on CD138^+^ or CD138^−^ populations and subsequently evaluating PE and APC expressions.

### Clinical specimens

Clinical bone marrow samples were obtained from five MM patients after informed. The study is approved by the National University of Singapore Institutional Review Board. Mononuclear cells were isolated by density gradient centrifugation using Histopaque-1077(Sigma-Aldrich, MO, USA). CD138^+^ and CD138^−^ populations were separated by using mouse antihuman CD138 antibody coupled to magnetic beads (Miltenyi Biotec, Germany). Resulting cells were depleted further for normal haematopoietic progenitor cells using mouse antihuman CD34 antibody (Miltenyi Biotec, Germany). The resulting cells were sorted using CD138 FITC, CD20 APC and CD27 PE antibodies[6,47] (BD PharMingen™, BD Biosciences, NJ, USA). CD138^−^CD20^+^CD27^+^ and CD138^−^CD20^−^CD27^+^ populations separated by FACS and the CD138^+^cells separated by MACS column were used to extract RNA for validation studies in clinical samples.

### Aldefluor Assay

Aldefluor assay was performed using the ALDEFLUOR^®^ Kit (Stem cell Technologies) as per the manufacturer's instructions. This assay measures the aldehyde dehydrogenase1 (ALDH1) enzymatic activity in the cells.

### Transplantation of MM cells into NOD/SCID/IL-2Rγnull (NOG) mice

Transplantation experiments using NOG mice were approved by National University Animal care and Use (AICUC) committee. NOG mice were bred and maintained in the university vivarium. Four to six weeks old mice were administered with Endoxan (150mg/Kg body weight/ day for 2 days; Cyclophosphamide, Baxter Oncology, GmBH, Germany) and rested for one day before the injection of cells. About 0.3-1 × 10^6^ cells/100μl of PBS (CD138^−^ or CD138^+^) or 100μl PBS were injected into dorsal tail vein. Mice were sacrificed when they exhibited symptoms including anorexia, lethargy, acute loss of weight and / or hind limb paralysis or at 14 -16 weeks in the absence of symptoms. Bone marrow and liver tissues were harvested, examined for the presence of tumors in the liver and human CD138^+^ cells in the bone marrow and liver tissues of sacrificed mice as evidence of engraftment.

### Immunohistochemistry

Liver tissues and bone marrow samples were processed and immuno detected as described in Supplementary information (M1).

### Gene expression profiling of CD138^−^ Vs CD138^+^ population

Total RNA was extracted from FACS separated CD138^+^ and CD138^−^ populations using RNeasy® Plus Mini Kit (Qiagen, GmBH) as per the manufacturer's instructions. Quality of the RNA samples was assessed by 260/280nm and 260/230nm absorption spectrum profiles and RNA Integrity Number (RIN). Gene expression profiling was carried out using Affymetrix human gene 1.0ST array chips as per the manufacturer's instructions. After hybridization and scanning, the raw data were imported into GeneSpring GX for normalization and identification of differentially expressed genes. Gene expression data was submitted to Gene Expression Omnibus (GEO Accession No: GGSE31305). Validation of the microarray data was done by qPCR of selected genes on cell lines and myeloma patient samples. About 300ng of total RNA from each sample was used for qPCR using Biorad iTaq Universal SYBR Green Supermix in a ABI-7500 fast real time PCR machine and fold change was calculated using 2ddC_T_ method, after normalizing to GAPDH. Western blot analyses were performed to verify the differential expression of PRC1component EZH2 and its downstream elements such as H3K27me3 and p16.

### Geneset Enrichment Analysis (GSEA)

Original Affymetrix HuGene v1.0ST array data of CD138^−^ and CD138^+^ cells were processed with RMA algorithm. GSEA analysis was performed using GSEA 2.0 program with 3 gene sets: leukemic stem cells (LSC), hematopoietic stem cells (HSC) and common progenitor cells (PC) extracted from original paper Nat. Med. 17:1086-1093, 2011 as target gene sets [48-50].

### Treatment of CD138^−^ and CD138^+^ populations with DZnep

Sorted CD138^+^ and CD138^−^ populations from both the cell lines were plated onto 96-well plates (3x 10^4^cells/ well) and treated with DZnep (0.5 mM/L) for 48 hrs and cell viability was assessed using Trypan blue dye exclusion. DMSO (Sigma-Aldrich, MO, USA) (0.1%) was used as a control. DZNep was kindly provided by Prof. Yu Qiang, Genome Institute of Singapore.

### Statistical analysis

Results are presented as the mean+/− standard deviation (SD). Comparisons between groups were performed using a 2-tailed, paired Student's *t* test and *P* values of < 0.05 were considered significant.

### Survival analysis

We analyzed the association of CD138^−^ signature and patient survival using two data sets: UAMS dataset (GSE2658) and Bortezomib dataset (GSE9782). Survival association was assessed by the Cox proportional hazard regression analysis using signature groups obtained by dividing signature index values into 4 equally spaced segments across the whole range. (Details: Supplementary information M2)

## Supplementary Tables, Figures and Information




