# Supplementary material for: Clonogenic Multiple Myeloma Cells have Shared stemness Signature Associated with Patient Survival
Source: Oncotarget. 2013 Jul 15;4(8):1230–40. doi: 10.18632/oncotarget.1145 (PMC3787153; doi:10.18632/oncotarget.1145)
Supplement: Supplementary file 2 [file oncotarget-04-1230-s002.pdf]

Supplementary data

Supplementary Table S3: Differentially expressed cell cycle associated genes*

Gene name	Description	Fold change (CD 138^{+} Vs CD138^{-})
CDKN1C	**Cyclin dependent kinase inhibitor 1C(p57)**	**+ 2.0666027**
CDKN1A	**Cyclin dependent kinase inhibitor 1A (p21)**	**+ 2.1310976**
CDK2	**Cyclin dependent Kinase 2**	**-2.0478663**
CDK4	**Cyclin dependent Kinase 4**	**-2.7181473**
CDK8	**Cyclin dependent Kinase 8**	**-2.292281**
CDK7	**Cyclin dependent Kinase 7**	**-2.107182**
CDKN2A	**Cyclin dependent kinase inhibitor 2A(p16)**	**-2.101037**
CDKN3	**Cyclin dependent kinase inhibitor 3**	**-3.0566034**
CCNE2	**Cyclin E2**	**-2.8048124**
CCNB2	**Cyclin B2**	**-2.5280573**
CCNC	**Cyclin C**	**-2.5377533**
CCNI	**Cyclin I**	**-2.0644908**
CCNG1	**Cyclin G1**	**-2.1613364**
CCNJL	**Cyclin J like**	**-2.0111609**
CCNB1IP	**Cyclin B1 interacting protein**	**-2.5442545**
CCNDBP	**Cyclin D type binding protein**	**-2.0553572**
CDC2	**Cell division cycle 2**	**-2.1113448**
CDC16	**Cell division cycle 16 homologue (S.*cerviciae*)**	**-2.3152206**
CDC23	**Cell division cycle 23 homologue (S.*cerviciae*)**	**-2.2285278**

Fold change 2.0 or more

Supplementary Table S4: list of primers used for q-PCR of selected PRC genes and their targets.

Genes	Primes
GMNN	Fp - GCCTTCTGCATCTGGATCTC<br>RP –TGACTCCTGGGTGACTCCTC
SUZ12	FP-CTG CCT CCA TTC GAA ACA TT<br>RP-AAC CAG GCT TGT TTT CCT GA
BMI	FP-ATG CAG CTC ATC CTT CTG CT<br>RP- CCG ATC CAA TCT GTT CTG GT
SATB1	FP-GATGCCCCTGATGCTACAGT<br>RP- CATTCCTCACTGTGGTGTGC
RYBP	FP- GACCAGCGAAACAAATCACA<br>RP- TCGATGAGGAGCGAGTCTTT
CCND2	FP-TGAGCTGCTGGCTAAGATCA<br>RP-ACGGTACTGCTGCAGGCTAT
EZH2	FP- TGATAGGGAAGCAGGGACTG<br>RP- CCGAGAATTTGCTTCAGAGG
EED	Fp- GAGAGGGAAGTGTCGACTGC<br>RP- GGTGTATCAGGGCGTTCAGT
BMP4	Fp- CTGGTCCACCACAATGTGAC<br>RP- CGATCGGCTAATCCTGACAT
BMP3	FP- AGGCCGTAGATCATTGTTGG<br>RP- CTGATACTGCACAGCCTCCA
BMP2	FP- GTCCTGAGCGAGTTCGAGTT<br>RP- AGTGCCTGCGATACAGGTCT
CDKN2C (P18)	FP- GGACCCAGGACTATCCCTTC<br>RP-TTTAGGGTCCCTTGTTCACG
NOTCH4	FP-CACGTGAACCCATGTGAGTC<br>RP- TTGAGCAGTTCTGTCCATCG
PAX4	FP-GAGGGTCTGGTTTTCCAACA<br>RP-TGCTGTGCAGAGATGATTCC
IGFBP1	Fp - CTGCGTGCAGGAGTCTGA<br>RP- GTACTGATGGCGTCCCAAAG

Fig S1.

(A) Heat map depicting enriched gens (H929 cell line)

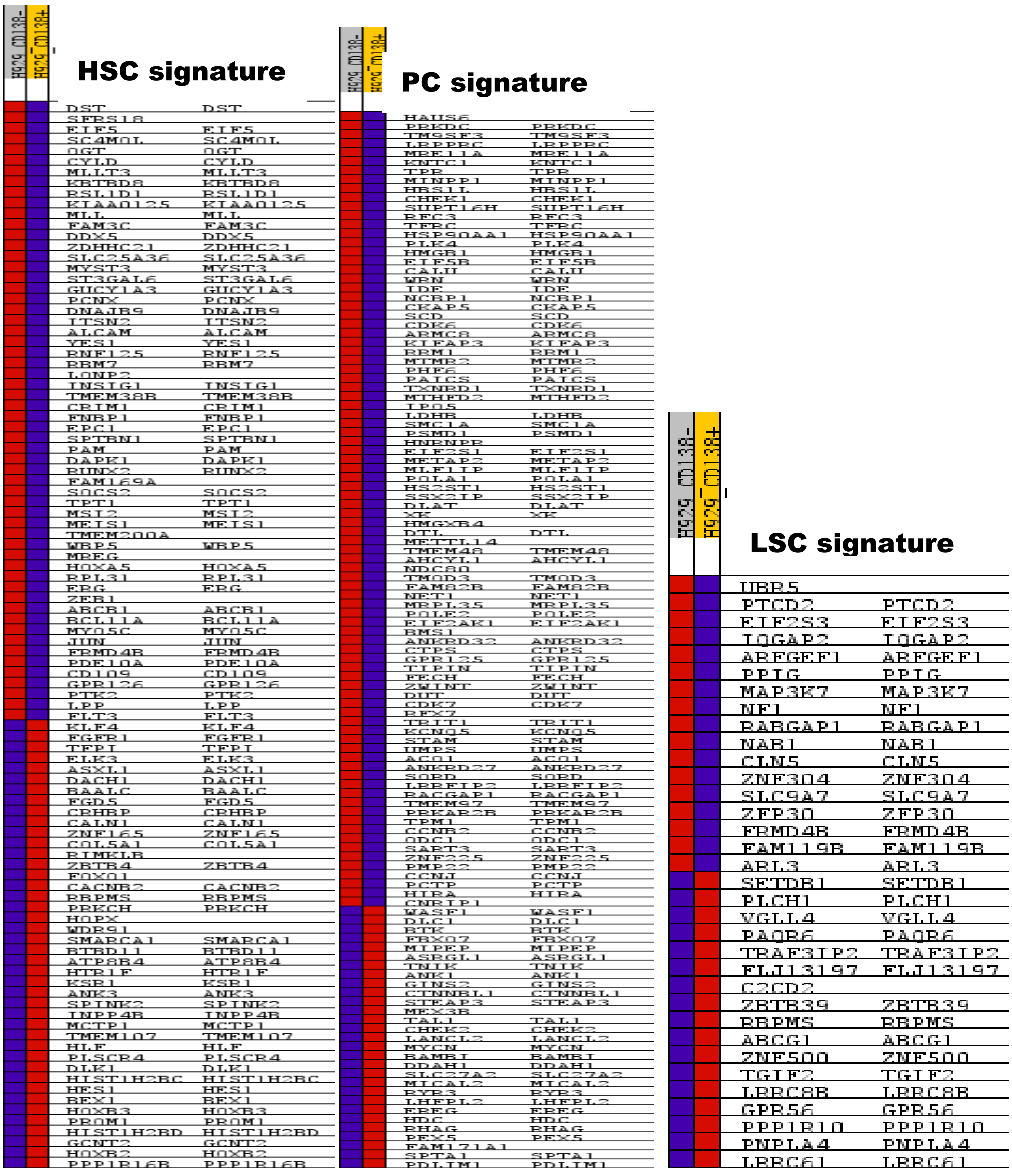

(B) Heat map depicting enriched gens (RPMI8226 cell line)

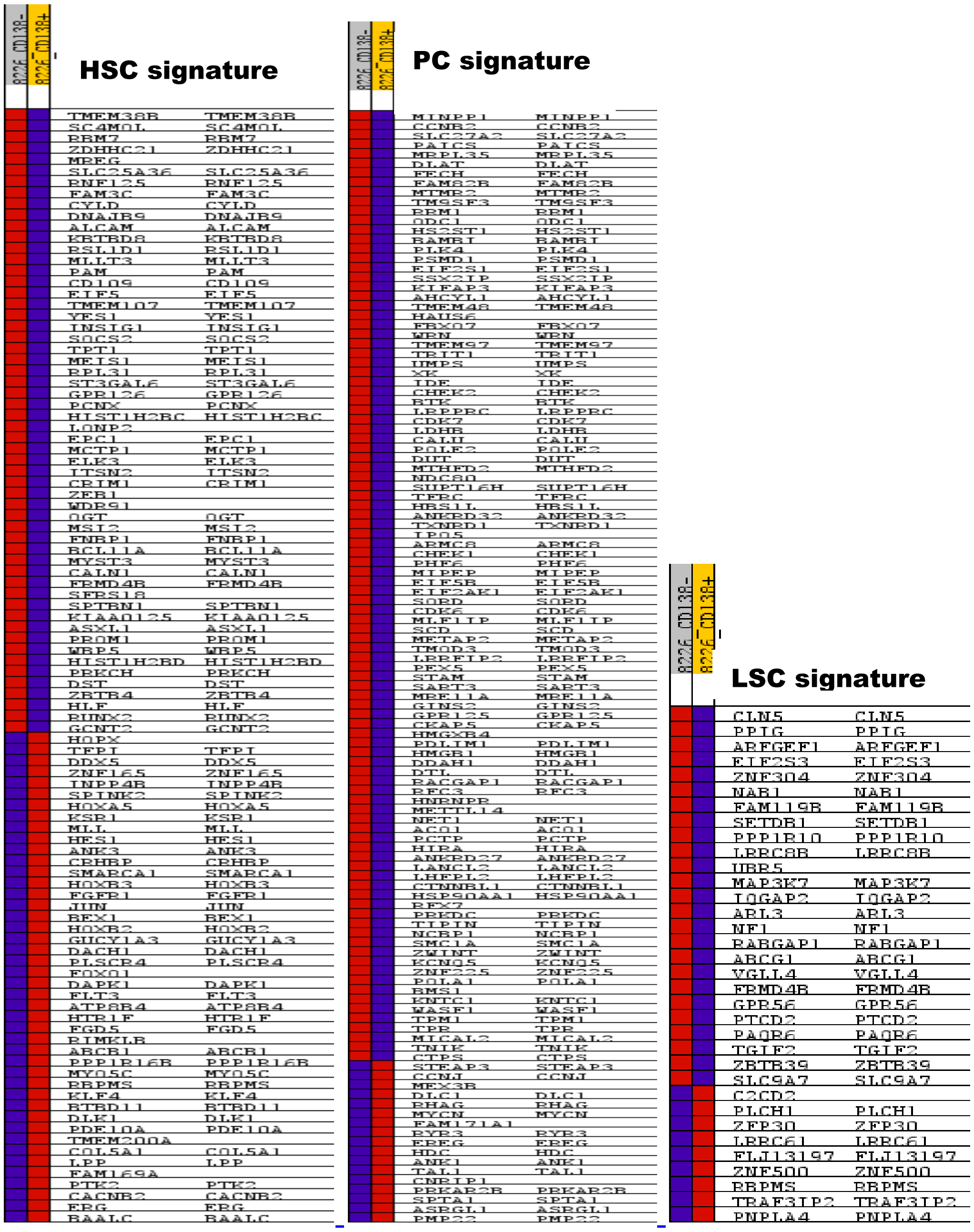

Figure S1. Heat map of lead genes of GSEA of CD138^{+} and CD138^{-} populations in H929 cells (A) and RMPI 8226 cells (B).

Fig S2.

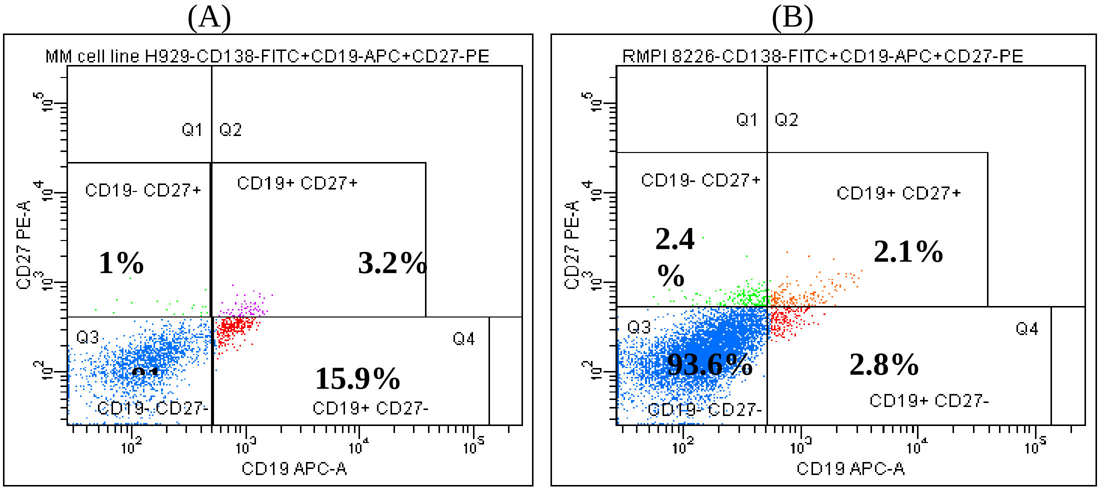

Figure S2. CD 138^{-} population is heterogeneous and represents different stages of B cell differentiation. Human myeloma cell lines H929 (A) or RPMI 8226 (B) were treated with antihuman CD138 FITC, CD27 PE and CD19 APC antibodies and subjected to flow cytometry. Percentage of each subset is depicted on the corresponding quarter for H929 and RPMI cell lines.

M1. Immuno-histochemistry Liver tissues harvested from the mice were fixed in 4% paraformaldehyde for overnight and then placed in Tissue-Tek biopsy uni-cassettes and embedded in wax using the Tissue-Tek tissue processor. After which, the tissues were sectioned into 4 µm longitudinal sections (Leica RM 2165). The sections were dipped in Histo-Clear (National Diagnistics, USA) for 5minutes twice and 30 seconds four times to remove the wax. The sections were then dipped twice in 100%, 95% and 75 % ethanol (1 min each) and subsequently rehydrated by immersing in water. The sections were then immersed in Dako Target Retrieval solution (citrate buffer pH6) and samples were heated to 110^{0}C for 10min in a T/T mega Multifunctional Histoprocessor. After the antigen retrieval step, 3% of H_2O_2 solution was added to the sections for 10min to quench the peroxidase activity of the tissue.. The tissue sections were then washed twice in water and once in Tris borate saline (TBS) and incubated with mouse

monoclonal antibody to human syndican-1 (Abcam, ab 82200) at 4 ^{0}C for overnight. After incubation the sections were washed for 5 min thrice in TBS and incubated with secondary anti mouse antibody, Dako Envision system with HRPO (Dako K4000) for 1hr at room temperature. After incubation, sections were washed in TBS thrice and immunodetection was done using liquid DAB substrate chromagen system (Dako K3468), visualized and imaged under Olympus CK 40 microscope.

Bone marrow: Bone marrow samples were resuspended in PBS and cell were then pelletted at 1000rpm for 3 min. The pellets were resuspended in RBC lysis buffer (8.36 g of NH_4Cl, 1g of $KHCO_3$, 0.04g of EDTA in 500 ml ddH_2O) and incubated at room temperature for about 30min. The cells were spun down and washed once with PBS and made into cell spots on glass slides using cytospin (Cytospin 4, Thermo Scientific). The cells were fixed using absolute methanol for 15 min and allowed to dry. Dried slides were stored at 4^{0}C until immunodetected and imaged

M2. Survival analysis of MM CD138^{-} signature using UAMS and Bortezomib datasets

Method

1. Associating Affymetrix HuGene v1 probes to HG-U133 Plus 2 probesets.

a. Duplicates of CD138- and CD138+ clones from MM cell lines 8226 and H929 (totally 8 chips of Affymetrix HuGene v1 platform) were processed with RMA. Fold changes in 8226 and H929 cell lines were estimated independently and those whose fold change values in 8226 and H929 were commonly over $2^{1.3}$ = 2.46 were selected for further analysis. At this step, 5323 and 358 probes were selected from cell lines 8226 and H929, respectively. As a whole, 169 probes were in common.

b. Associated genes for those probes were searched from NetAffx annotation using EntrezGene id as the key. At this step, 93 of them were not associated with any gene but 74 of them were associated with unique gene and 2 with 2 genes.

c. Probesets of Affymetrix HG-U133 Plus 2 platform were looked for those uniquely matched genes. At this step, 72 of the genes had associated probesets in HG-U133 Plus 2 platform; 14 of them were associated with unique probesets; 18 with 2 probesets; 40 with more than 2 probesets.

d. For multiply associated genes, unique association was established by choosing those with maximum median expression in MMRC reference sample gene expression data.

2. Survival association.

a. MAS5 preprocessed gene expression profiling data for UAMS dataset (GSE2658) and Bortezomib dataset (GSE9782) were normalized with probeset-wise median normalization; for each probeset, we first determined its median MAS5 intensity level over all samples and expression value is estimated by the logarithm (base 2) of MAS5 intensity divided by median level (log2ratio).

b. Expression measure of a sample was calculated as the median of log2ratio for signature probesets.

c. Survival association was assessed by the Cox proportional hazard regression analysis using signature groups obtained by dividing signature index values into 4 equally spaced segments across the whole range. The number of samples in each segment was as follows: UAMS dataset: 51 (top range), 445 (2nd range), 56 (3rd range), and 7 (bottom range); Bortezomib dataset: 18 (top range), 91 (2nd range), 70 (3rd range), 9 (bottom range).
